# Spirituality and religiosity in children, adolescents and their families in a vulnerable context: a scoping review

**DOI:** 10.1590/0034-7167-2023-0425

**Published:** 2024-11-22

**Authors:** Marcela Teixeira de Souza, Layane Cristina Araújo, Alexandre Ernesto Silva, Liana Amorim Corrêa Trotte, Elaine Cristina Rodrigues Gesteira

**Affiliations:** I Universidade Federal do Rio de Janeiro. Rio de Janeiro, Rio de Janeiro, Brazil; II Universidade Federal de São João del-Rei. Divinópolis, Minas Gerais, Brazil

**Keywords:** Spirituality, Child, Adolescent, Religion, Social Vulnerability, Espiritualidad, Niño, Adolescente, Religión, Vulnerabilidad Social

## Abstract

**Objective:**

to map evidence in the literature on the spirituality and religiosity of children, adolescents and their families in social vulnerability.

**Methods:**

this is a scoping review based on the JBI methodology, with the search without delimiting the time period, in English, Portuguese and Spanish, in the Virtual Health Library, PubMed, Embase, Cochrane Library, Scopus and Web of Science databases.

**Results:**

twenty-two studies were identified. The most studied population were adolescents, followed by children and their families. Regarding the setting, the context of vulnerability related to the low socioeconomic level experienced by these populations was highlighted. Furthermore, spirituality and religiosity were considered important for coping, social support, purpose and strength.

**Conclusion:**

there is an influence of spirituality and religiosity in the lives of children, adolescents and families, being a protective factor and a source of comfort, playing essential tools for living in context.

## RESUMEN

**Objetivo:**

mapear la evidencia en la literatura sobre la espiritualidad y religiosidad de niños, adolescentes y sus familias en el contexto de vulnerabilidad social.

**Métodos:**

se trata de una revisión de alcance basada en la metodología JBI, con búsqueda sin delimitar el período de tiempo, en inglés, portugués y español, en la Biblioteca Virtual en Salud, PubMed, Embase, Biblioteca Cochrane, Scopus y Web of Science.

**Resultados:**

se identificaron 22 estudios. La población más estudiada fueron los adolescentes, seguidos de los niños y sus familias. En cuanto al escenario, se destacó el contexto de vulnerabilidad relacionado con el bajo nivel socioeconómico que experimentan estas poblaciones. Además, la espiritualidad y la religiosidad se consideraron importantes para afrontar la situación, el apoyo social, el propósito y la fortaleza.

**Conclusión:**

existe influencia de la espiritualidad y la religiosidad en la vida de niños, adolescentes y familias, siendo un factor protector y fuente de consuelo, jugando herramientas esenciales para vivir en contexto.

## INTRODUCTION

It is known that the development of children and adolescents goes through several stages over the years and that they are experienced in different social contexts. In this regard, several authors have already demonstrated the relationship between social vulnerability, well-being, mental health and school participation of children and adolescents^(1)^.

Parallel to this, there is an increase in the number of studies that mention that spirituality and religiosity can be used as therapeutic resources for healthcare professionals, especially nursing professionals, and as tools to guide the care of individuals in society^(2)^. Research aimed at healthcare, as carried out by doctors and nurses, highlights that spirituality can provide spiritual well-being, strength, comfort, security and promote connection with oneself, with nature and with family members^(2,3)^. Furthermore, there are authors who consider that spirituality is present from the beginning of life, thus influencing everything from the fetal stage to childhood and adulthood^(4)^.

Spirituality is understood as a person’s connection with the sacred and the transcendent, in the face of existential issues that involve human life^(5,6)^. In particular, in the childhood phase, spirituality can be observed through speech, gestures, gaze, expression and communication, with such actions being associated with this child’s behavior and evolution^(4)^.

On the other hand, religiosity deals with specific religious practices guided by religious institutions through dogmas^(2-6)^. These activities are based on doctrines, rituals and traditions that enable the transformation of attitudes and experiences in each individual’s life^(7)^.

In situations that involve human development, spiritual and social dimensions are directly related to the environment in which people live^(4)^. In the meantime, the concept of vulnerability is understood as the possibility of human beings experiencing a certain context. Furthermore, there are authors who state the existence of situations in which an individual may necessarily be experiencing some deprivation, such as poverty, illness and distress that causes harm, and these are considered vulnerable individuals^(8)^.

The definition of social vulnerability is considered from a multifactorial phenomenon that can involve conditions of extreme poverty, deprivation of education and access to health, child and adolescent violence, life-threatening diseases and drug abuse^(9,10)^. In the meantime, people who experience vulnerable scenarios are exposed to risks and demographic and/or socioeconomic aspects that can directly influence coexistence, availability of opportunities, development and social relations of a population^(10)^.

Given the social scenario and based on its multidimensional notion, more and more individuals suffer due to differences, whether social, economic, or cultural^(11)^. Furthermore, based on the multidimensional notion of vulnerable contexts, people have restricted access to necessary services and do not have protection from the State, influencing their quality of life and well-being^(10)^.

Therefore, the present study is justified given the scarcity of studies and the relevance of investigating the influence of spirituality and religiosity in the different social contexts of children, adolescents and families in vulnerable situations to support reflections that can direct multidisciplinary actions in the care of these children, adolescents and families.

## OBJECTIVE

To map evidence of spirituality and religiosity of children, adolescents and their families in the context of social vulnerability in the literature.

## METHODS

### Ethical aspects

This research was not submitted for consideration by the Research Ethics Committee, as it is a scoping review, whose data are secondary and available in the literature. However, copyright, analysis, referencing and sharing of study results were respected.

### Study design

This is a scoping review study based on the assumptions of the review methodology presented by JBI, used to explore, expand and clarify the main evidence present in the literature about a given field of research^(12)^. The review protocol was registered on the Open Science Framework (OSF) platform, with DOI 10.17605/OSF.IO/AR4EB.

### Methodological procedures

Bibliographic search explored national and international literature, which enabled analysis and synthesis of already published studies. The research question was constructed based on the Population, Context and Concept (PCC) strategy, which defined: P - children and adolescents aged 0 to 18; C - spirituality, religiosity; C - vulnerability situation. Given this, the question defined for the review was: what is the influence of spirituality and religiosity on children, adolescents and their families in a context of social vulnerability?

A broad search was carried out in the OSF and databases to identify reviews with a similar theme. From this, the steps to carry out the scoping review followed.

Subsequently, the descriptors available in the Virtual Health Library, National Library of Medicine (PubMed), Scopus, Web of Science (WoS), Latin American and Caribbean Literature in Health Sciences (LILACS), Embase and Cochrane Library databases were selected. Finally, the bibliographic survey took place from March to April 2023. Furthermore, it was decided that, in case of disagreement, a third researcher would be called.

In the combinations chosen for the final selection of articles, the sets of terms were associated: religiosity, spirituality, child and adolescent, family and social vulnerability. It is noteworthy that, in this study, the social vulnerability criterion is operationalized as a person or community affected in their ability to cope with natural situations or those caused by another individual^(8-10)^.

Furthermore, the Boolean terms AND and OR were used to assist in searching the databases, generating the strategies presented in Chart 1.

**Chart 1:** Crossing of descriptor sets in databases

Database	Search expression
VHL	(*Espiritualidade* OR Spirituality OR *Espiritualidad* OR *Spiritualité* OR *Religião* OR Religion OR *Religión*) AND (*Criança* OR Child OR *Niño* OR *Enfant* OR *Adolescente* OR Adolescent OR *Família* OR Family OR *Familia* OR *Famille* OR Teenager) AND (“*Vulnerabilidade Social*” OR “Social Vulnerability” OR “*Vulnerabilidad Social*” OR “*Vulnérabilité Sociale*” OR *Poverty* OR Poverty OR *Pauvreté*)
PubMed, Scopus, Web of Science, LILACS, Embase, Cochrane Library	(Spirituality OR Religion) AND (Child OR Adolescent OR Family OR Teenager) AND (“Social Vulnerability” OR Poverty)

### Data collection and organization

Bibliographic survey was carried out by two researchers independently, following the steps proposed by the scoping review^(12)^. However, there were no disagreements among researchers at the time of independent analysis and reading.

Manuscripts in English, Portuguese and Spanish, addressing children and adolescents aged 0 to 19 in socially vulnerable contexts as well as their respective families were included. Furthermore, studies that dealt with spirituality and religiosity as well as their applicability in therapeutic care were included. Editorials, abstracts, correspondence, monographs and reviews were excluded. The time frame of studies was not estimated.

For data collection, an instrument was developed that included authors, year, country, study objective, study design, population and main outcomes, to extract data from manuscripts. Articles were also assessed regarding the level of evidence, according to the criteria developed by Galvão (2006)^(13)^.

The scientific texts identified in the databases were exported to the Qatar Computing Research Institute’s Rayyan application to be analyzed according to the inclusion or exclusion of texts^(14)^. After reading titles and abstracts, only 57 articles remained to be assessed for possible inclusion. Of these studies, after reading in full, those that did not correspond to the objective were excluded, obtaining the final number of 22 texts included in the present review.

**Figure 1:**
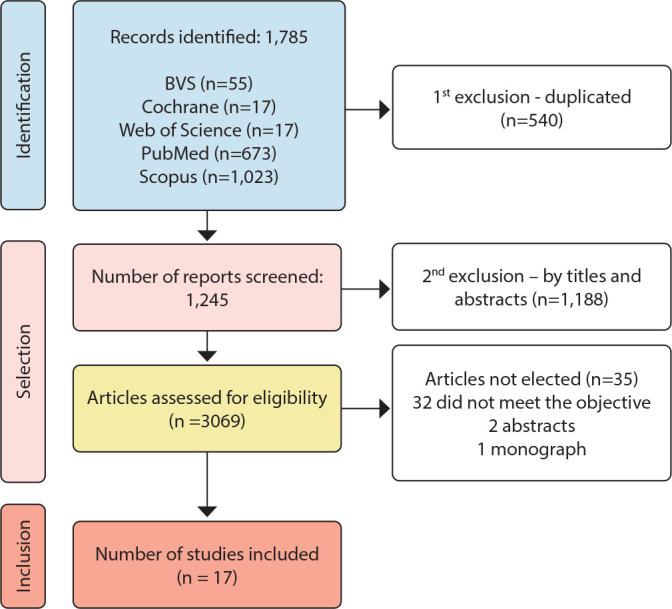
Scoping review search flowchart

For the set of articles used, the collection instrument was used to gather the relevant data to be reproduced in this manuscript. To better visualize the methodological path, Figure 1 was created based on the Preferred Reporting Items for Systematic reviews and Meta-Analyses extension for Scoping Reviews (PRISMA-ScR)^(15)^.

## RESULTS

Of the 22 articles selected, one was published in 2022, four in 2021, three in 2020, four in 2019, three in 2018, one in 2017, two in 2014, one in 2013, one in 2012, one in 2011, one in 2010, one in 2008, one in 2007, two in 2004, one in 2000, and one in 1993. The results also demonstrate that such studies were from countries such as Africa (n=7), USA (n=7), Brazil (n=1), Israel (n=2), Dominican Republic (n=1), New Zealand (n=1), El Salvador (n= 1), Bangladesh (n=1) and Malta (n=1).

Data on author, year, country, study objective, population, main outcomes and level of evidence (Chart 2) and information regarding the population and study setting (Chart 3) were extracted to prepare the chart.

**Chart 2:** Presentation of studies regarding authors, year of publication, country of research, objective and study design, main outcomes and level of evidence

Title	Author/year/ country	Study objective	Study design	Main outcomes	Level of evidence
The impact of caring and connectedness on adolescent health and well-being^(16)^	Resnick MD *et al*./1993/USA	Identify protective factors against silently disturbed and acting out adolescents’ behaviors.	Quantitative	Measures of caregiving and connection outperformed demographic variables such as two-parent versus single-parent family structure as protective factors against high-risk behaviors.	IV
A questionnaire to measure factors that protect youth against stressors of inner-city life^(17)^	Weist MD *et al*./2000/USA	Report the development of a questionnaire that protects inner-city youth against stressors such as poverty, crime and violence.	Qualitative	The religious involvement factor was considered important for the development of urban young people.	IV
Religious beliefs, faith community involvement and depression: a study of rural, low-income mothers^(18)^	Garrison ME *et al*./2004/USA	Investigate the connection between religion and mental health in 131 low-income rural mothers.	Quantitative	Religious beliefs regarding involvement in the faith community were negatively related to depressive symptoms, indicating that mothers with stronger religious beliefs and more involvement in religious activities may experience fewer depressive symptoms.	IV
A qualitative Exploration of Resilience in Pre-Adolescent AIDS Orphans Living in a Residential Care Facility^(19)^	Pienaar A *et al*./2011/Africa	Identify and investigate assets that operate in children’s lives to help them cope with exposure to adversity.	Qualitative and exploratory	Morality, social values, religion and faith helped children define their purpose in life.	IV
Maternal religious attendance and low birth weight^(20)^	Burdette AM *et al*./2012/USA	Test whether maternal religious attendance protects against low birth weight among predominantly African-American, lower socioeconomic status, and single women.	Cohort	Maternal religious attendance is protective against low birth weight. It was also associated with lower cigarette use and poor nutrition.	III
In response to community violence: coping strategies and involuntary stress responses among Latino adolescentes^(21)^	Epstein-Ngo Q *et al*./2013/ Dominican Republic	Investigate how stress coping strategies can mediate and moderate the relationship between exposure to violence and psychological well-being.	Quantitative	The results indicated that religious coping was a significant moderator of the relationship between personal victimization and depression.	IV
Dynamics of oppression and coping from traumatology perspective: The example of Palestinian adolescents^(22)^	Kira IA *et al*./2014/Israel	Test a model of trauma and coping for West Bank adolescents.	Quantitative	Religiosity had a direct association with social support and forgiveness, also causing a significant reduction in depression.	IV
Search for Transcendence Revealed in Childhood Narratives of Poverty, Abuse and Neglect, and Social Isolation^(23)^	Rogers CL/2014/USA	Analyze reports of children in situations of poverty, neglect, abuse and social isolation.	Reflection	There was a need to hear reports from children to recognize and respond more intentionally to spiritual hunger when considering childhood experiences.	IV
Personal encounters with children in an informal settlement: Exploring spirituality^(24)^	Kruidenier R *et al*./2017/Africa	Explore the factors that contribute to the spiritual development of children living in informal settlements in Africa.	Reflection	It was found that the faith community, daycare center and other partners played a crucial role in children’s spiritual development.	IV
Family Rituals in Low-Income African American Families at Risk for Trauma Exposure and Associations with Toddlers’ Regulation of Distress^(25)^	Bocknek EL/2018/South Africa	Assess the relationship between family rituals and coping with distress.	Qualitative	There was a significant association between spirituality and regulation of babies’ distress.	IV
Patterns of Spiritual Connectedness during Adolescence: Links to Coping and Adjustment in Low-Income Urban Youth^(26)^	Wright AW *et al*./2018/Africa	Determine the profile of changes in spiritual connection over time in a population of low-income adolescents.	Quantitative	Three distinct profiles emerged: low and stable, moderate with declines during the study and high and stable. Adolescents in the high and stable profile showed more goal orientation, life satisfaction, emotion management and more effective coping strategies.	IV
*Resiliência familiar: percepção de mães em situação de pobreza* ^(27)^	Matos LAS *et al*./2018/Brazil	Understand the perception of family resilience from the point of view of mothers in situations of poverty.	Qualitative descriptive	Despite the risks associated with poverty, protective intra-family factors that generate benefits for human development were observed, such as an optimistic outlook, spirituality, hope, social support from family and neighbors, teamwork, support in conflict resolution, family unity and social and economic resources through social benefits, such as *Bolsa Família* (Family Allowance).	IV
Measuring Spirituality, Hope, and Thriving Among Salvadoran Youth: Initial Findings from the Compassion International Study of Positive Youth Development^(28)^	Tirrell JM *et al*./2019/El Salvador	Test measures of spirituality, hope and success among two groups of young people.	Cross-sectional	Young people enrolled in the Compassion International organization reported higher levels of transcendence (spirituality).	IV
Trauma coping of mothers and children among poor people in Haiti: Mixed methods study of community-level research^(29)^	Roysircar G *et al*./2019/USA	Investigate how Haitian children are socialized by their mothers’/caregivers’ religious and non-religious coping in the context of ongoing trauma.	Mixed-methods	Correlations and multiple regressions examined the relationships of the frequencies of mothers’ coping themes with their children’s scores on two factor dimensions.	IV
Maternal and Family Correlates of Intrinsic Religiosity Profiles Among Low-Income Urban African American Adolescents^(30)^	Kliewer W *et al*./2020/Africa	Examine the contributions of maternal religiosity and the family emotional climate in distinguishing adolescents’ intrinsic religiosity.	Quantitative	Analyzes revealed that maternal religious attendance and commitment differentiated young people who had high levels of intrinsic religiosity (41%) from young people who had low levels of religiosity.	IV
Religion, Health, Social Capital and Place: The Role of the Religious, Social Processes and the Beneficial and Detrimental Effects on the Health and Wellbeing of Inhabitants in Deprived Neighbourhoods in Malta^(31)^	Satariano B/2020/Malta	Emphasize the important role that place plays in determining how religious social processes operate and impact health and well-being.	Qualitative descriptive	It was found that faith and religious practices can have a positive impact on health and well-being.	IV
Shared spiritual beliefs between adolescents with cancer and their families^(32)^	Livingston J/2020/USA	Assess the sharing of spiritual beliefs among adolescents with cancer and their families.	Controlled randomized	Family members may not share spiritual beliefs with adolescents and may be unaware of the importance of spiritual well-being for adolescents.	II
Assessing sources of resilience in orphans and vulnerable children in Amajuba District schools^(33)^	Lawrence KC *et al*./2021/Africa	Assess sources of resilience in orphaned and vulnerable children in district schools in local communities.	Mixed-methods	Religion and spirituality were considered sources of resilience, serving as an inner strength to remain resilient despite vulnerability.	IV
It Would be Harder Without Faith”: An Exploratory Study of Low-Income Families’ Experiences of Early Childhood Inclusive Education in New Zealand^(34)^	Zhang KC *et al*./2021/New Zealand	Assess, from parents’ perspective, the experience of inclusive early childhood education in low-income families.	Exploratory	Participants’ religion and faith allowed them to have a positive view of negative experiences and to have resilience.	IV
Prevalence and associated factors of depression among adolescent boys and girls in Bangladesh: findings from a nationwide survey^(35)^	Mridha MK *et al*./2021/Bangladesh	Assess the prevalence and factors associated with depression among adolescent boys and girls.	Cross-sectional	Only among girls, the Muslim religion was associated with depression.	IV
Qualitative, longitudinal exploration of coping strategies and factors facilitating infant and young child feeding practices among mothers in rural Rwanda^(36)^	Ahishakiye J *et al*./2021/Rwanda	Explore coping strategies that facilitate appropriate breastfeeding and complementary feeding practices among rural Rwandan mothers from birth to 1 year of children’s life.	Qualitative descriptive	Personal factors such as breastfeeding self-efficacy, religious beliefs, and perceived benefits of breastfeeding were among the facilitating factors.	IV
Material Deprivation and Subjective Poverty Association With Subjective Well-Being Reported by Children: Religiosity as a Protective Factor^(37)^	Gross-Manos D *et al*./2022/Israel	Explore the association between material deprivation and subjective poverty with subjective well-being as well as the possible moderating effect of religiosity.	Descriptive	The findings suggest that religiosity plays a protective role.	IV

**Chart 3:** Characterization of selected studies according to population and context

Title	Population (n)	Context
The impact of caring and connectedness on adolescent health and well-being^(16)^	Adolescents (36,254)	Public schools
A questionnaire to measure factors that protect youth against stressors of inner-city life^(17)^	Adolescents (256)	Poverty
Religious beliefs, faith community involvement and depression: a study of rural, low-income mothers^(18)^	Mothers (131)	Low income
A qualitative Exploration of Resilience in Pre-Adolescent AIDS Orphans Living in a Residential Care Facility^(19)^	Children (8)	Life-threatening disease and poverty
Maternal religious attendance and low birth weight^(20)^	Mothers of children (4,898)	Low socioeconomic status
In response to community violence: coping strategies and involuntary stress responses among Latino adolescents^(21)^	Adolescents (223)	Poverty
Dynamics of oppression and coping from traumatology perspective: The example of Palestinian adolescents^(22)^	Adolescents (438)	Oppression and poverty
Search for Transcendence Revealed in Childhood Narratives of Poverty, Abuse and Neglect, and Social Isolation^(23)^	Children (53)	Poverty and negligence
Personal encounters with children in an informal settlement: Exploring spirituality^(24)^	Children (8)	Poverty
Family Rituals in Low-Income African American Families at Risk for Trauma Exposure and Associations with Toddlers’ Regulation of Distress^(25)^	Families (75) Children aged 24 to 30 months	Low socioeconomic status
Patterns of Spiritual Connectedness during Adolescence: Links to Coping and Adjustment in Low-Income Urban Youth^(26)^	Adolescents (355)	Low income
*Resiliência familiar: percepção de mães em situação de pobreza* ^(27)^	Families (16)	Poverty
Measuring Spirituality, Hope, and Thriving Among Salvadoran Youth: Initial Findings from the Compassion International Study of Positive Youth Development^(28)^	Young people (888) aged 9-15	Poverty
Trauma coping of mothers and children among poor people in Haiti: Mixed methods study of community-level research^(29)^	Mothers (27) of children (42)	Poverty
Maternal and Family Correlates of Intrinsic Religiosity Profiles Among Low-Income Urban African American Adolescents^(30)^	Mothers and adolescents (326)	Low income
Religion, Health, Social Capital and Place: The Role of the Religious, Social Processes and the Beneficial and Detrimental Effects on the Health and Wellbeing of Inhabitants in Deprived Neighbourhoods in Malta^(31)^	Families (20)	Poverty
Shared spiritual beliefs between adolescents with cancer and their families^(32)^	Adolescents (126)	Life-threatening disease
Assessing sources of resilience in orphans and vulnerable children in Amajuba District schools^(33)^	Children (303)	Poverty
It Would be Harder Without Faith”: An Exploratory Study of Low-Income Families’ Experiences of Early Childhood Inclusive Education in New Zealand^(34)^	Parents of children with disabilities or chronic illnesses (30)	Disability/chronic illness
Prevalence and associated factors of depression among adolescent boys and girls in Bangladesh: findings from a nationwide survey^(35)^	Adolescents (9,856)	Subnormal agglomerations and rural areas
Qualitative, longitudinal exploration of coping strategies and factors facilitating infant and young child feeding practices among mothers in rural Rwanda^(36)^	Mothers (17)	Poverty
Material Deprivation and Subjective Poverty Association With Subjective Well-Being Reported by Children: Religiosity as a Protective Factor^(37)^	Children (2,773)	Poverty and material deprivation

Studies were assessed according to the criteria established by Galvão, which classifies studies into: level I - systematic review or meta-analysis; level II - randomized clinical trial; level III - clinical trial without randomization; level IV - cohort and case-control studies; level V - descriptive and qualitative studies; level VI - single descriptive and qualitative study; level VII - opinion of authorities and/or experts.

## DISCUSSION

From the results found, it is possible to observe that the presence of spirituality and religiosity in the context of the lives of children, adolescents and their families plays a role as a source of resilience and inner strength to deal with the difficulties arising from vulnerable settings^(33)^.

The geographical distribution of included articles appears to be wide, however, the number of Brazilian studies represents less than half of the research carried out in countries such as Africa and the USA, drawing attention to this scoping review, as it is a developing country, where the majority of the population considers themselves Christian, presenting regional disparities and evident social inequalities. Thus, greater emphasis on Brazilian studies on the association between spirituality, religiosity and social vulnerability would be expected.

Furthermore, the number of studies carried out in countries located in Africa totaled more than 50% of the studies carried out. This result may be related to the social context and human development, lack of access to public services, food, housing and violence. Vulnerable groups are those disproportionately exposed to risks, with members changing dynamically. Policy responses can transform individuals from initially non-vulnerable to vulnerable, due to loss of income or lack of support^(38)^.

It is clear that, given scenarios with social vulnerability, it is necessary to understand the guiding causes and conditions that impede the exercise of citizenship. In this way, it is understood that the vulnerable human condition is associated both with the specific situations of each individual and also with collective ones^(39)^.

In turn, when relating the low economic level of a country, as evidenced in some regions of Africa, it is understood that the low-income population is harmed or restricted from accessing tools that guarantee the achievement of basic fundamental rights. In view of this, the lack of resources and lack of access to public services, such as health and education, can directly impact child and youth development^(40)^.

Furthermore, with regard to the education of these socially vulnerable children and adolescents, such as when faced with a diagnosis of chronic diseases, authors emphasize professors’ unpreparedness to intervene in such situations and minimize student problems. Therefore, subsidies and knowledge to help overcome the difficulties encountered are necessary to ensure and promote social protection^(40)^.

In this research, most of selected studies are related to adolescents and the different contexts of vulnerability they experience, such as low-income scenarios, violence, drug consumption, life-threatening illnesses and traumas. Thus, there are authors who state that, regarding coping tools for young people, spirituality and religiosity can provide preventive aspects for drug use, promoting well-being, self-efficiency, self-respect and self-control for adolescents. Therefore, the transformative effect on the experience of these individuals in the face of the uncertainties and changes that occur in transition from childhood to adolescence can be noted^(41)^.

As can be seen, the increase in studies carried out over the years, demonstrated in this review, demonstrates that more and more research is being developed on spirituality and religiosity in contexts of social vulnerability. In the meantime, such research demonstrates that spirituality and religiosity are used as tools for making positive daily changes in the face of problems and adversities.

In particular, other authors^(42)^ note that spirituality and religiosity must also be observed in families, given that it is important to consider their influencing role in their children’s lives, thus promoting positive coping strategies.

From this perspective, it was seen that belief and faith are essential sources of comfort and support for coping with stress for adolescents with cancer, generated by the process of becoming ill, being characterized as strategies that provide a source of comfort^(43)^. Another study that analyzes the impact of spirituality and religiosity on the use of psychoactive substances by adolescents showed that both function as protective and informative factors, in addition to enabling future perspectives, strengthening the bond and presence of family, friends, social groups and contact with the environment^(9)^.

Spirituality is capable of providing confidence, reflection, meaning in life, self-knowledge and direction in adolescents’ decision-making^(44)^. For families and caregivers of children in vulnerable health settings, it is possible to observe the use of positive religious-spiritual coping by these people who present a coping strategy used in the face of the experiences of childhood chemotherapy treatment^(45)^.

From another perspective, it can be observed that religion is a faith resource for adolescents, which enables appreciation of life, achievement of satisfaction, hope and transcendence, and, in this way, acts as a protective source in these adolescents’ daily lives^(46)^.

More recently, a study portrayed the positive influence of religiosity on the mental health, physical health and well-being of Egyptian adolescents, showing that it is a significant agent for this population^(7)^. Thus, the impact of religiosity on adolescents in Spain can also be seen, demonstrating that more than 30% of young people mentioned that their religious beliefs directly impact their actions and that the high level of religiosity of individuals influences the lower consumption of alcoholic beverages and contributes as a modifying factor in the healthcare process of this vulnerable population who considered religion necessary in their lives^(47)^.

In the family environment, it has been demonstrated that spirituality and religiosity can also be used as protective tools for families living in situations of poverty, low education, inadequate housing and violence. In this way, through faith, beliefs, optimistic vision and hope, families of children in situations of social vulnerability overcome adversities, stressful situations and conflicts arising from economic, social and educational problems^(48)^.

Regarding the context of health conditions, the relevance of discussing the benefits of spirituality and religiosity in the care offered to patients and their families by the multidisciplinary team is observed. A recent study showed that religiosity and spirituality are essential dimensions in the process of healing, rehabilitation and overcoming, and that they must be implemented in the science of care by healthcare professionals^(48)^.

On the other hand, another recent survey confirmed that the lack of preparation of many health team professionals to deal with the spiritual situations of patients, families and caregivers is remarkable. Furthermore, it highlighted the need to incorporate the theme into professional training and qualification, with the aim of improving quality of care in terms of the relevance of individuals’ spiritual and religious values^(49)^.

In this context, in relation to the relevance of the subject for undergraduate students, a study investigated the perception of nursing students about spirituality, being evident that there is an understanding of the importance of the relationship between spirituality and care in nursing care, considering the humanistic, holistic and spiritual/religious perspectives^(50)^.

The Brazilian Society of Family Medicine corroborates by expanding its vision on the approach to spiritual assistance by the multidisciplinary team, highlighting that spiritual assistance strengthens the bond and connection with patients, since trust and the feeling of attention to needs are improved from this relationship^(51)^.

In this sense, the term “evidence-based spirituality” emphasizes that spiritual and religious activities positively influence numerous aspects of human health^(52)^. Therefore, it is necessary for more and more new studies to be carried out to expand knowledge about the need for spiritual and religious care in vulnerable scenarios, which compromise the well-being of children, adolescents and their families. Studies are also needed to expand the positive evidence of offering spiritual support by healthcare professionals.

Therefore, by understanding the complexities of vulnerable groups and spirituality and religiosity as tools that can be used, it is necessary to implement and use such subsidies by and for these populations. In turn, through the implementation of spiritual care in the education and health sectors, professionals will be able to plan and adopt strategies to assist such groups.

### Study limitations

The number of databases searched may have contributed to limiting access to other data. Another limiting factor was the context researched, in which only one Brazilian study was identified. It is believed that this article contributes to the development of future research, especially in Latin America, considering that only two articles were found.

### Contributions to nursing, health, or public policy

The study developed allows us to identify the strengths and weaknesses regarding the spirituality and religiosity of children, adolescents and their families in a vulnerable context, which makes it possible to implement measures in the training of healthcare professionals so that there is a spiritual/religious approach to the patient in order to guarantee holistic and comprehensive care.

## FINAL CONSIDERATIONS

This scoping review contributed to demonstrating that there is an influence of religiosity and spirituality in the lives of children, adolescents and families, and this was portrayed as a protective factor, source of comfort and resilience tool in facing adversity in a vulnerable context.

Therefore, investigating how the presence of spirituality and religiosity can influence the health-disease process becomes essential for the implementation of comprehensive care for children, adolescents, families and communities, given the need to consider the biopsychosocial and spiritual context in which individuals are included.
