# Supplementary material for: Spirituality and religiosity in children, adolescents and their families in a vulnerable context: a scoping review
Source: Rev Bras Enferm. 2024 Nov 22;77(5):e20230425. doi: 10.1590/0034-7167-2023-0425 (PMC11653882; doi:10.1590/0034-7167-2023-0425)
Supplement: Translation

# Translation: Espiritualidade e religiosidade em crianças, adolescentes e suas famílias em um contexto vulnerável: revisão de escopo

**DOI:** 10.1590/0034-7167-2023-0425pt

**Authors:** Marcela Teixeira de Souza, Layane Cristina Araújo, Alexandre Ernesto Silva, Liana Amorim Corrêa Trotte, Elaine Cristina Rodrigues Gesteira

**Affiliations:** I Universidade Federal do Rio de Janeiro. Rio de Janeiro, Rio de Janeiro, Brasil; II Universidade Federal de São João del-Rei. Divinópolis, Minas Gerais, Brasil

**Keywords:** Espiritualidade, Criança, Adolescente, Religião, Vulnerabilidade Social

## Abstract

**Objetivo:**

mapear na literatura as evidências sobre a espiritualidade e religiosidade de crianças, adolescentes e suas famílias no contexto de vulnerabilidade social.

**Métodos:**

trata-se de revisão de escopo baseada na metodologia do JBI, com a busca sem delimitar o período temporal, nos idiomas inglês, português e espanhol, nas bases de dados Biblioteca Virtual em Saúde, PubMed, Embase, *Cochrane Library*, Scopus e *Web of Science*.

**Resultados:**

foram identificados 22 estudos. A população mais estudada foi de adolescentes, seguida de crianças e suas famílias. No que tange ao cenário, foi destacado o contexto de vulnerabilidade relacionada ao baixo nível socioeconômico vivenciado por essas populações. Além disso, a espiritualidade e a religiosidade foram consideradas importantes para enfrentamento, apoio social, propósito e força.

**Conclusão:**

há influência da espiritualidade e religiosidade na vida de crianças, adolescentes e familiares, sendo fator de proteção e fonte de conforto, desempenhando ser ferramentas essenciais para a vivência em contexto.

## INTRODUÇÃO

Sabe-se que o desenvolvimento de crianças e adolescentes perpassa por diversas etapas ao longo dos anos e que são vivenciadas em diferentes contextos sociais. Nesse sentido, diversos autores já demonstram a relação entre vulnerabilidade social, bem-estar, saúde mental e participação escolar de crianças e adolescentes^(1)^.

Paralelo a isso, observa-se o crescimento da realização de estudos que mencionam que a espiritualidade e a religiosidade podem ser utilizadas como recursos terapêuticos para os profissionais de saúde, em especial de enfermagem, e como ferramentas para nortear o cuidado dos indivíduos na sociedade^(2)^. Pesquisas visando ao cuidado em saúde, como realizadas por médicos e enfermeiros, destacam que a espiritualidade pode proporcionar bem-estar espiritual, força, conforto, segurança e promover conexão consigo, com a natureza e com os familiares^(2,3)^. Além disso, há autores que consideram que a espiritualidade está presente desde o início da vida, influenciando, desse modo, desde a fase fetal até a infantil e a adulta^(4)^.

Entende-se por espiritualidade a conexão de uma pessoa com o sagrado e o transcendente, diante de questões existenciais que envolvem a vida humana^(5,6)^. Em especial, na fase infantil, a espiritualidade pode ser observada por meio da fala, dos gestos, do olhar, da expressão e da comunicação, sendo tais ações associadas ao comportamento e à evolução dessa criança^(4)^.

Por outro lado, a religiosidade trata de práticas religiosas específicas norteadas por instituições religiosas através dos dogmas^(2-6)^. Essas atividades são fundamentadas em doutrinas, rituais e tradições que possibilitam a transformação de atitudes e vivências na vida de cada indivíduo^(7)^.

Diante de situações que implicam o desenvolvimento humano, as dimensões espiritual e social estão diretamente relacionadas ao ambiente em que as pessoas vivem^(4)^. Nesse ínterim, o conceito de vulnerabilidade é compreendido como a possibilidade de o ser humano experimentar determinado contexto. Outrossim, há autores que afirmam a existência de situações em que um indivíduo pode estar necessariamente vivenciando alguma privação, como a pobreza, a doença e um sofrimento que cause prejuízo, sendo esses considerados indivíduos vulnerados^(8)^.

A definição de vulnerabilidade social é considerada a partir de um fenômeno multifatorial que pode envolver condições de extrema pobreza, privação da educação e acesso à saúde, violência infantojuvenil, doenças ameaçadoras da vida e abuso de drogas^(9,10)^. Nesse ínterim, pessoas que vivenciam cenários vulneráveis estão expostas a riscos e aspectos demográficos e/ou socioeconômicos que podem influenciar diretamente o convívio, a disponibilidade de oportunidades, o desenvolvimento e as relações sociais de uma população^(10)^.

Diante do cenário social e a partir de sua noção multidimensional, cada vez mais indivíduos sofrem devido às diferenças, sejam sociais, econômicas, ou culturais^(11)^. Além disso, a partir da noção multidimensional dos contextos vulneráveis, pessoas possuem restrição de acesso a serviços necessários e não possuem proteção do Estado, influenciando sua qualidade de vida e bem-estar^(10)^.

Sendo assim, o presente estudo justifica-se diante da escassez de estudos e da relevância de investigar a influência da espiritualidade e da religiosidade nos diferentes contextos sociais de crianças, adolescentes e famílias em situações de vulnerabilidade, para subsidiar reflexões que possam direcionar ações multiprofissionais no cuidado dessas crianças, adolescentes e famílias.

## OBJETIVO

Mapear na literatura as evidências da espiritualidade e da religiosidade de crianças, adolescentes e suas famílias no contexto de vulnerabilidade social.

## MÉTODOS

### Aspectos éticos

A presente pesquisa não foi submetida à apreciação do Comitê de Ética e Pesquisa, por se tratar de revisão de escopo, cujos dados são secundários e disponíveis na literatura. Contudo, respeitaram-se os direitos autorais, a análise, o referenciamento e o compartilhamento dos resultados do estudo.

### Tipo de estudo

Trata-se de estudo de revisão de escopo, com base nos pressupostos da metodologia de revisão apresentada pelo JBI, utilizada para explorar, expandir e esclarecer as principais evidências presentes na literatura acerca de um determinado campo de pesquisa^(12)^. O protocolo da revisão foi registrado na plataforma *Open Science Framework* (OSF), com DOI 10.17605/OSF.IO/AR4EB.

### Procedimentos metodológicos

A busca bibliográfica explorou a literatura nacional e internacional, que possibilitou análise e síntese de estudos já publicados. A pergunta de pesquisa foi construída com base na estratégia População, Contexto e Conceito (PCC), em que se definiu: P - crianças e adolescentes de 0 a 18 anos; C - espiritualidade, religiosidade; C - cenário de vulnerabilidade. Diante disso, a pergunta delimitada para a revisão foi: qual a influência da espiritualidade e da religiosidade em crianças, adolescentes e suas famílias em um contexto de vulnerabilidade social?

Foi realizada uma ampla busca na OSF e em bases de dados para identificação das revisões com temática similar. A partir disto, seguiu-se com as etapas para a realização da revisão de escopo.

Em seguida, foi realizada a seleção dos descritores disponíveis nas bases de dados Biblioteca Virtual em Saúde, *National Library of Medicine* (PubMed), Scopus, *Web of Science* (WoS), Literatura Latino-Americana e do Caribe em Ciências da Saúde (LILACS), Embase e *Cochrane Library*. Por fim, o levantamento bibliográfico ocorreu no período de março a abril de 2023. Além disso, definiu-se que, em caso de divergência, um terceiro pesquisador seria acionado.

Nas combinações eleitas para a seleção final dos artigos, foi realizada a associação dos conjuntos de termos: religiosidade, espiritualidade, criança e adolescente, família e vulnerabilidade social. Ressalta-se que, neste estudo, operacionaliza-se o critério de vulnerável social como pessoa ou comunidade afetada em sua capacidade de enfrentamento diante de situações naturais ou causadas por outro indivíduo^(8-10)^.

Além disso, empregaram-se os termos booleanos *AND* e *OR* para auxiliar nas buscas nas bases de dados, gerando as estratégias apresentadas no Quadro 1.

**Quadro 1:** Cruzamento dos conjuntos de descritores nas bases de dados

Base de dados	Expressão de busca
BVS	(Espiritualidade *OR Spirituality OR Espiritualidad OR Spiritualité OR Religião OR Religion OR Religión*) *AND* (Criança *OR Child OR Niño OR Enfant OR* Adolescente *OR Adolescent OR* Família *OR Family OR Familia OR Famille OR Teenager*) *AND* (“Vulnerabilidade Social” *OR* “*Social Vulnerability*” *OR* “*Vulnerabilidad Social*” *OR* “*Vulnérabilité Sociale*” *OR* Pobreza *OR Poverty OR Pauvreté*)
PubMed, Scopus, *Web of Science*, LILACS, Embase, *Cochrane Library*	(*Spirituality OR Religion*) *AND* (*Child OR Adolescent OR Family OR Teenager*) *AND* (“*Social Vulnerability*” *OR Poverty*)

### Coleta e organização dos dados

O levantamento bibliográfico foi realizado por dois pesquisadores de forma independente, obedecendo às etapas propostas pela revisão de escopo^(12)^. No entanto, não houve divergências entre os pesquisadores no momento da análise e leitura independente.

Foram incluídos manuscritos no idioma inglês, português e espanhol, com abordagem de crianças e adolescentes na faixa etária de 0 a 19 anos em contextos de vulnerabilidade social, bem como as respectivas famílias. Além disso, foram incluídos estudos que tratavam sobre espiritualidade e religiosidade, bem como a sua aplicabilidade no cuidado terapêutico. Foram excluídos editoriais, resumos, correspondências, monografias e resenhas. Não foi estimado o recorte temporal de publicações.

Para a coleta de dados, foi elaborado um instrumento que contemplava autores, ano, país, objetivo do estudo, tipo de estudo, população e principais desfechos, para a realização da extração de dados dos manuscritos. Os artigos também foram avaliados quanto ao nível de evidência, de acordo com os critérios elaborados por Galvão (2006)^(13)^.

Os textos científicos identificados nas bases de dados foram exportados para o aplicativo Rayyan do *Qatar Computing Research Institute* para serem analisados conforme a inclusão ou exclusão dos textos^(14)^. Após a leitura do título e resumo, restaram apenas 57 artigos a serem avaliados para a possível inclusão. Desses estudos, após leitura na íntegra, foram excluídos aqueles que não correspondiam ao objetivo, obtendo o número final de 22 textos incluídos na presente revisão.

Para o conjunto dos artigos utilizados, utilizouse o instrumento de coleta para reunir os dados relevantes a serem reproduzidos neste manuscrito. Para melhor visualização do percurso metodológico, foi confeccionada a Figura 1 baseada na ferramenta *Preferred Reporting Items for Systematic reviews and Meta-Analyses extension for Scoping Reviews* (PRISMA-ScR)^(15)^.

**Figura 1:** Fluxograma de busca da revisão de escopo

## RESULTADOS

Dos 22 artigos selecionados, um foi publicado em 2022, quatro, em 2021, três, em 2020, quatro, em 2019, três, em 2018, um, em 2017, dois, em 2014, um, em 2013, um, em 2012, um, em 2011, um, em 2010, um, em 2008, um, em 2007, dois, em 2004, um, em 2000, e um, em 1993. Os resultados também demonstram que tais publicações foram de países como África (n=7), EUA (n=7), Brasil (n=1), Israel (n=2), República Dominicana (n=1), Nova Zelândia (n=1), El Salvador (n=1), Bangladesh (n=1) e Malta (n=1).

Foram extraídos os dados sobre autor, ano, país, objetivo do estudo, população, principais desfechos e nível de evidência (Quadro 2) e informações referentes à população e ao cenário dos estudos (Quadro 3) para a elaboração do quadro.

**Quadro 2:** Apresentação dos estudos quanto aos autores, ano de publicação, país da pesquisa, objetivo e tipo de estudo, principais desfechos e nível de evidência

Título	Autor/ano/ país	Objetivo do estudo	Tipo de estudo	Principais desfechos	Nível de evidência
*The impact of caring and connectedness on adolescent health and well-being* ^(16)^	Resnick MD *et al*./1993/EUA	Identificar fatores de proteção contra os comportamentos silenciosamente perturbados e atuantes de adolescentes.	Quantitativo	As medidas de cuidado e conexão superaram as variáveis demográficas, como estrutura familiar biparental *versus* monoparental como fatores de proteção contra comportamentos de alto risco.	IV
*A questionnaire to measure factors that protect youth against stressors of inner-city life* ^(17)^	Weist MD *et al*./2000/EUA	Relatar o desenvolvimento de um questionário que proteja os jovens do centro da cidade contra estressores, como pobreza, crime e violência.	Qualitativo	O fator envolvimento religioso foi considerado importante para o desenvolvimento dos jovens urbanos.	IV
*Religious beliefs, faith community involvement and depression: a study of rural, low-income mothers* ^(18)^	Garrison ME *et al*./2004/EUA	Investigar a conexão entre religião e saúde mental de 131 mães rurais de baixa renda.	Quantitativo	As crenças religiosas quanto ao envolvimento na comunidade de fé foram negativamente relacionadas aos sintomas depressivos, indicando que mães com crenças religiosas mais fortes e mais envolvimento em atividades religiosas podem apresentar menos sintomas depressivos.	IV
*A qualitative Exploration of Resilience in Pre-Adolescent AIDS Orphans Living in a Residential Care Facility* ^(19)^	Pienaar A *et al*./2011/África	Identificar e investigar ativos que operam na vida das crianças para ajudá-las a lidar com a exposição a adversidades.	Qualitativo e exploratório	A moralidade, os valores sociais, a religião e a fé ajudaram as crianças na definição do propósito de vida.	IV
*Maternal religious attendance and low birth weight* ^(20)^	Burdette AM *et al*./2012/EUA	Testar se a frequência religiosa materna protege contra o baixo peso ao nascer entre mulheres predominantemente afro-americanas, de nível socioeconômico mais baixo e solteiras.	Coorte	A frequência religiosa materna é protetora contra o baixo peso ao nascer. Também foi associada ao menor uso de cigarros e má nutrição.	III
*In response to community violence: coping strategies and involuntary stress* *responses among Latino adolescentes* ^(21)^	Epstein-Ngo Q *et al*./2013/República Dominicana	Investigar como as estratégias de enfrentamento ao estresse podem mediar e moderar a relação entre a exposição à violência e o bem-estar psicológico.	Quantitativo	Os resultados indicaram que o *coping* religioso foi um moderador significativo da relação entre vitimização pessoal e depressão.	IV
*Dynamics of oppression and coping from traumatology perspective: The example of Palestinian adolescents* ^(22)^	Kira IA *et al*./2014/Israel	Testar um modelo de trauma e enfrentamento por adolescentes da Cisjordânia.	Quantitativo	A religiosidade teve associação direta no apoio social com o perdão, ocasionando também a diminuição significativa da depressão.	IV
*Search for Transcendence Revealed in Childhood Narratives of Poverty, Abuse and Neglect, and Social Isolation* ^(23)^	Rogers CL/2014/EUA	Analisar relato de crianças em situação de pobreza, negligência, abuso e isolamento social.	Reflexão	Houve necessidade de ouvir relato de crianças para reconhecer e responder mais intencionalmente à fome espiritual na consideração das experiências da infância.	IV
*Personal encounters with children in an informal settlement: Exploring spirituality* ^(24)^	Kruidenier R *et al*./2017/África	Explorar os fatores que contribuem para o desenvolvimento espiritual das crianças que vivem no assentamento informal na África.	Reflexão	Verificou-se que a comunidade de fé, a creche e outros parceiros desempenharam um papel crucial no desenvolvimento espiritual das crianças.	IV
*Family Rituals in Low-Income African American Families at Risk for Trauma Exposure and Associations with Toddlers’ Regulation of Distress* ^(25)^	Bocknek EL/2018/África do Sul	Avaliar a relação entre rituais familiares e o enfrentamento do sofrimento.	Qualitativo	Houve associação significativa entre a espiritualidade e a regulação do sofrimento dos bebês.	IV
*Patterns of Spiritual Connectedness during Adolescence: Links to Coping and Adjustment in Low-Income Urban Youth* ^(26)^	Wright AW *et al*./2018/África	Determinar o perfil de mudanças na conexão espiritual ao longo do tempo em uma população de adolescentes de baixa renda.	Quantitativo	Emergiram três perfis distintos, baixo e estável, moderado, com declínios durante o estudo e alto e estável. Os adolescentes no perfil alto e estável evidenciaram mais direcionamento para os objetivos, satisfação com a vida, gerenciamento de emoções e estratégias de enfrentamento mais eficazes.	IV
Resiliência familiar: percepção de mães em situação de pobreza^(27)^	Matos LAS *et al*./2018/Brasil	Compreender a percepção acerca da resiliência familiar sob o ponto de vista de mães em situação de pobreza.	Descritivo qualitativo	Apesar dos riscos associados à pobreza, observaram-se fatores intrafamiliares protetivos geradores de benefícios ao desenvolvimento humano, tais como visão otimista, espiritualidade, esperança, apoio social familiar e nos vizinhos, trabalho em equipe, apoio na resolução de conflitos, união familiar e recursos sociais e econômicos através de benefícios sociais, como o Bolsa Família.	IV
*Measuring Spirituality, Hope, and Thriving Among Salvadoran Youth: Initial Findings from the Compassion International Study of Positive Youth Development* ^(28)^	Tirrell JM *et al*./2019/El Salvador	Testar medidas de espiritualidade, esperança e sucesso entre dois grupos de jovens.	Transversal	Os jovens matriculados na organização *Compassion International* relataram níveis mais altos de transcendência (espiritualidade).	IV
*Trauma coping of mothers and children among poor people in Haiti: Mixed methods study of community-level research* ^(29)^	Roysircar G *et al*./2019/EUA	Investigar como as crianças haitianas são socializadas pelo enfrentamento religioso e não religioso de suas mães/cuidadores no contexto de trauma contínuo.	Misto	Correlações e regressões múltiplas examinaram as relações das frequências dos temas de enfrentamento das mães com as pontuações de seus filhos em duas dimensões fatoriais.	IV
*Maternal and Family Correlates of Intrinsic Religiosity Profiles Among Low-Income Urban African American Adolescents* ^(30)^	Kliewer W *et al*./2020/África	Examinar as contribuições da religiosidade materna e do clima emocional familiar na distinção de religiosidade intrínseca de adolescentes.	Quantitativo	As análises revelaram que a frequência e o compromisso religioso materno diferenciam os jovens que tinham altos níveis de religiosidade intrínseca (41%) dos jovens que tinham baixos níveis de religiosidade.	IV
*Religion, Health, Social Capital and Place: The Role of the Religious, Social Processes and the Beneficial and Detrimental Effects on the Health and Wellbeing of Inhabitants in Deprived Neighbourhoods in Malta* ^(31)^	Satariano B/2020/Malta	Enfatizar o importante papel que o lugar tem na determinação de como os processos sociais religiosos operam e impactam a saúde e o bem-estar.	Descritivo qualitativo	Verificou-se que a fé e as práticas religiosas podem gerar um impacto positivo na saúde e no bem-estar.	IV
*Shared spiritual beliefs between adolescents with cancer and their families* ^(32)^	Livingston J/2020/EUA	Avaliar o compartilhamento de crenças espirituais entre adolescentes com câncer e suas famílias.	Randomizado controlado	Os familiares podem não compartilhar crenças espirituais com os adolescentes e podem desconhecer a importância do bem-estar espiritual para os adolescentes.	II
*Assessing sources of resilience in orphans and vulnerable children in Amajuba District schools* ^(33)^	Lawrence KC *et al*./2021/África	Avaliar as fontes de resiliência em crianças órfãs e vulneráveis em escolas distritais das comunidades locais.	Misto	A religião e a espiritualidade foram consideradas fontes de resiliência, servindo como força interior para permanecerem resilientes apesar da vulnerabilidade.	IV
*It Would be Harder Without Faith”: An Exploratory Study of Low-Income Families’ Experiences of Early Childhood Inclusive Education in New Zealand* ^(34)^	Zhang KC *et al*./2021/Nova Zelândia	Avaliar, na perspectiva dos pais, a experiência de educação inclusiva na primeira infância de famílias de baixa renda.	Exploratório	A religião e a fé dos participantes lhes permitiam ter uma visão positiva sobre as experiências negativas e ter resiliência.	IV
*Prevalence and associated factors of depression among adolescent boys and girls in Bangladesh: findings from a nationwide survey* ^(35)^	Mridha MK *et al*./2021/Bangladesh	Avaliar a prevalência e os fatores associados à depressão entre meninos e meninas adolescentes.	Transversal	Apenas entre meninas, a religião muçulmana foi associada à depressão.	IV
*Qualitative, longitudinal exploration of coping strategies and factors facilitating infant and young child feeding practices among mothers in rural Rwanda* ^(36)^	Ahishakiye J *et al*./2021/Ruanda	Explorar as estratégias de enfrentamento que facilitam práticas apropriadas de amamentação e alimentação complementar entre mães rurais de Ruanda desde o nascimento até 1 ano de vida da criança.	Descritivo qualitativo	Fatores pessoais, como autoeficácia na amamentação, crenças religiosas e benefícios percebidos da amamentação, estavam entre os fatores facilitadores.	IV
*Material Deprivation and Subjective Poverty Association With Subjective Well-Being Reported by Children: Religiosity as a Protective Factor* ^(37)^	Gross-Manos D *et al*./2022/Israel	Explorar a associação entre privação material e pobreza subjetiva com o bem-estar subjetivo, bem como o possível efeito moderador da religiosidade.	Descritivo	Os achados sugerem que a religiosidade desempenha um papel protetor.	IV

**Quadro 3:** Caracterização dos estudos selecionados segundo a população e o contexto

Título	População (n)	Contexto
*The impact of caring and connectedness on adolescent health and well-being* ^(16)^	Adolescentes (36.254)	Escolas públicas
*A questionnaire to measure factors that protect youth against stressors of inner-city life* ^(17)^	Adolescentes (256)	Pobreza
*Religious beliefs, faith community involvement and depression: a study of rural, low-income mothers* ^(18)^	Mães (131)	Baixa renda
*A qualitative Exploration of Resilience in Pre-Adolescent AIDS Orphans Living in a Residential Care Facility* ^(19)^	Crianças (8)	Doença ameaçadora à vida e pobreza
*Maternal religious attendance and low birth weight* ^(20)^	Mães de crianças (4.898)	Baixo nível socioeconômico
*In response to community violence: coping strategies and involuntary stress* *responses among Latino adolescents* ^(21)^	Adolescentes (223)	Pobreza
*Dynamics of oppression and coping from traumatology perspective: The example of Palestinian adolescents* ^(22)^	Adolescentes (438)	Opressão e pobreza
*Search for Transcendence Revealed in Childhood Narratives of Poverty, Abuse and Neglect, and Social Isolation* ^(23)^	Crianças (53)	Pobreza e negligência
*Personal encounters with children in an informal settlement: Exploring spirituality* ^(24)^	Crianças (8)	Pobreza
*Family Rituals in Low-Income African American Families at Risk for Trauma Exposure and Associations with Toddlers’ Regulation of Distress* ^(25)^	Famílias (75) Crianças de 24 a 30 meses	Baixo nível socioeconômico
*Patterns of Spiritual Connectedness during Adolescence: Links to Coping and Adjustment in Low-Income Urban Youth* ^(26)^	Adolescentes (355)	Baixa renda
Resiliência familiar: percepção de mães em situação de pobreza^(27)^	Famílias (16)	Pobreza
*Measuring Spirituality, Hope, and Thriving Among Salvadoran Youth: Initial Findings from the Compassion International Study of Positive Youth Development* ^(28)^	Jovens (888) de 9-15 anos	Pobreza
*Trauma coping of mothers and children among poor people in Haiti: Mixed methods study of community-level research* ^(29)^	Mães (27) de crianças (42)	Pobreza
*Maternal and Family Correlates of Intrinsic Religiosity Profiles Among Low-Income Urban African American Adolescents* ^(30)^	Mães e adolescentes (326)	Baixa renda
*Religion, Health, Social Capital and Place: The Role of the Religious, Social Processes and the Beneficial and Detrimental Effects on the Health and Wellbeing of Inhabitants in Deprived Neighbourhoods in Malta* ^(31)^	Famílias (20)	Pobreza
*Shared spiritual beliefs between adolescents with cancer and their families* ^(32)^	Adolescentes (126)	Doença ameaçadora à vida
*Assessing sources of resilience in orphans and vulnerable children in Amajuba District schools* ^(33)^	Crianças (303)	Pobreza
*It Would be Harder Without Faith”: An Exploratory Study of Low-Income Families’ Experiences of Early Childhood Inclusive Education in New Zealand* ^(34)^	Pais de filhos com deficiência ou doença crônica (30)	Deficiência/doença crônica
*Prevalence and associated factors of depression among adolescent boys and girls in Bangladesh: findings from a nationwide survey* ^(35)^	Adolescentes (9.856)	Aglomerados subnormais e zonas rurais
*Qualitative, longitudinal exploration of coping strategies and factors facilitating infant and young child feeding practices among mothers in rural Rwanda* ^(36)^	Mães (17)	Pobreza
*Material Deprivation and Subjective Poverty Association With Subjective Well-Being Reported by Children: Religiosity as a Protective Factor* ^(37)^	Crianças (2.773)	Pobreza e privação material

Os estudos foram avaliados segundo os critérios estabelecidos por Galvão, que classifica os estudos em: nível I - revisão sistemática ou metanálise; nível II - ensaio clínico randomizado; nível III - ensaio clínico sem randomização; nível IV - estudos de coorte e caso-controle; nível V - estudos descritivos e qualitativos; nível VI - estudo descritivo e qualitativo único; nível VII - opinião de autoridades e/ou especialistas.

## DISCUSSÃO

A partir dos resultados encontrados, é possível observar que a presença de espiritualidade e religiosidade no contexto de vida de crianças, adolescentes e seus familiares exerce um papel de fonte de resiliência e força interior para lidarem com as dificuldades decorrentes de cenários de vulnerabilidade^(33)^.

A distribuição geográfica dos artigos incluídos revela ser ampla, entretanto o quantitativo de publicações brasileiras representa menos da metade das pesquisas realizadas em países como a África e os EUA, chamando a atenção nesta revisão de escopo, pois trata-se de um país em desenvolvimento, em que a maioria da população se considera cristã, apresentando disparidades regionais e evidentes desigualdades sociais. Nesse sentido, seria esperado maior destaque de estudos brasileiros sobre a associação entre espiritualidade, religiosidade e vulnerabilidade social.

Além disso, a quantidade de estudos realizados em países localizados na África totalizou mais de 50% das publicações realizadas. Tal resultado pode estar relacionado ao contexto social e desenvolvimento humano, carência de acesso a serviços públicos, alimentação, moradia e violência. Grupos vulneráveis são aqueles expostos desproporcionalmente a riscos, com membros mudando dinamicamente. As respostas políticas podem tornar indivíduos inicialmente de não vulneráveis a vulneráveis, devido à perda de renda ou falta de apoio^(38)^.

Percebe-se que, diante dos cenários com vulnerabilidade social, é necessário compreender as causas norteadoras e as condições que impedem o exercício da cidadania. Desse modo, entende-se que a condição humana vulnerável está associada tanto às situações específicas de cada indivíduo quanto também às coletivas^(39)^.

Por sua vez, ao relacionar o baixo nível econômico de um país, como evidenciado em algumas regiões da África, entende-se que a população com baixa renda é prejudicada ou é restrita a acessar ferramentas que garantam a conquista dos direitos fundamentais básicos. Em vista disso, a falta de recursos e a escassez de acesso aos serviços públicos, como a saúde e a educação, podem impactar diretamente o desenvolvimento infantojuvenil^(40)^.

Além disso, no que se refere à educação dessas crianças e adolescentes em vulnerabilidade social, como diante de diagnóstico de doenças crônicas, autores enfatizam o despreparo dos professores em intervir em tais situações e minimizar os problemas dos alunos. Desse modo, subsídios e conhecimento para auxiliar na superação diante das dificuldades encontradas são necessários para assegurar e promover a proteção social^(40)^.

Na presente pesquisa, a maioria dos estudos selecionados possui relação com os adolescentes e os diferentes contextos de vulnerabilidade vivenciados, como cenários de baixa renda, violência, consumo de drogas, doenças ameaçadoras à vida e traumas. Dessa forma, há autores que afirmam que, no que tange às ferramentas de enfrentamento para os jovens, a espiritualidade e a religiosidade podem proporcionar aspectos preventivos para o uso de drogas, promoção de bem-estar, autoeficiência, autorrespeito e autocontrole para os adolescentes. Nota-se, portanto, o efeito transformador na vivência desses indivíduos diante das incertezas e mudanças que ocorrem na transição da fase infantil para a adolescência^(41)^.

Como pode-se perceber, o aumento das publicações realizadas ao longo dos anos, demonstradas nesta revisão, demonstra que cada vez mais pesquisas estão sendo desenvolvidas sobre espiritualidade e religiosidade em contextos de vulnerabilidade social. Nesse ínterim, tais pesquisas demonstram que a espiritualidade e a religiosidade são utilizadas como ferramentas para a realização de mudanças cotidianas positivas em diante dos problemas e adversidades.

Em especial, outros autores^(42)^ observam que a espiritualidade e a religiosidade devem ser observadas também nas famílias, haja vista que se torna importante considerar o papel influenciador delas na vida dos filhos, promovendo, assim, estratégias de enfrentamento positivas.

Nessa perspectiva, foi visto que a crença e a fé são fontes essenciais de conforto e apoio para o enfrentamento do estresse por adolescentes com câncer, gerado pelo processo de adoecer, sendo caracterizadas como estratégias de fonte de conforto^(43)^. Outro estudo que analisa o impacto da espiritualidade e religiosidade no uso de substâncias psicoativas por adolescentes evidenciou que ambas funcionam como fatores protetores e de informação, além de possibilitar perspectivas de futuro, fortalecimento do vínculo e presença da família, amigos, grupo social e contato com o ambiente^(9)^.

A espiritualidade é capaz de proporcionar confiança, reflexão, sentido de vida, autoconhecimento e direcionamento diante da tomada de decisão de adolescentes^(44)^. Para as famílias e cuidadores de crianças em cenários vulnerados de saúde, observa-se a utilização do *coping* religioso-espiritual positivo por essas pessoas que apresentam uma estratégia de enfrentamento utilizada diante das experiências vivenciadas pelo tratamento quimioterápico infantojuvenil^(45)^.

Sob outra perspectiva, pode-se observar que a religião é um recurso de fé para adolescentes, o qual possibilita a valorização da vida, o alcance da satisfação, a esperança e a transcendência, e, dessa forma, age como fonte protetiva no cotidiano desses adolescentes^(46)^.

Mais recentemente, estudo retratou a influência positiva da religiosidade na saúde mental, saúde física e bem-estar de adolescentes egípcios, evidenciando ser um agente significativo para essa população^(7)^. Assim, pode-se perceber também o impacto da religiosidade em adolescentes da Espanha, demonstrando que mais de 30% dos jovens mencionaram que suas crenças religiosas impactam diretamente suas ações e que o alto nível de religiosidade dos indivíduos influencia o menor consumo de bebidas alcoólicas e contribui como fator modificador no processo de cuidado em saúde dessa população vulnerada que considerava necessária a religião em suas vidas^(47)^.

No ambiente familiar, foi demonstrado que a espiritualidade e a religiosidade também podem ser utilizadas como ferramentas protetoras diante de famílias que vivem em situação de pobreza, baixa escolaridade, moradias inadequadas e violência. Dessa forma, por meio da fé, crenças, visão otimista e esperança, famílias de crianças em situação de vulnerabilidade social superam adversidades, situações estressantes e conflitos oriundos de problemas econômicos, sociais e de educação^(48)^.

No que tange ao contexto de condição de saúde, observa-se a relevância acerca da discussão sobre os benefícios da espiritualidade e religiosidade na assistência oferecida ao paciente e sua família pela equipe multiprofissional. Recente estudo evidenciou que a religiosidade e a espiritualidade são dimensões essenciais no processo de cura, reabilitação e superação, e que devem ser implementadas na ciência do cuidado pelos profissionais de saúde^(48)^.

Por outro lado, outra recente pesquisa ratificou que é notável o despreparo de muitos profissionais da equipe de saúde para lidar com as situações espirituais dos pacientes, familiares e cuidadores. Além disso, evidenciou a necessidade da incorporação da temática na formação e qualificação profissional, com o intuito de melhorar a qualidade da assistência no que tange à relevância dos valores espirituais e religiosos do indivíduo^(49)^.

Nesse contexto, em relação à relevância do assunto para graduandos, estudo investigou a percepção de estudantes de enfermagem acerca da espiritualidade, sendo evidenciado que há a compreensão da importância da relação entre espiritualidade e cuidado na assistência de enfermagem, considerando a perspectiva humanista, holística e espiritual/religiosa^(50)^.

A Sociedade Brasileira de Medicina de Família corrobora ao ampliar sua visão sobre a abordagem da assistência espiritual pela equipe multidisciplinar, destacando que a assistência espiritual fortalece o vínculo e a conexão com o paciente, uma vez que a confiança e a sensação de atenção às necessidades são melhoradas a partir dessa relação^(51)^.

Nesse sentido, o termo “espiritualidade baseada em evidências” enfatiza que as atividades espirituais e religiosas influenciam positivamente inúmeros aspectos da saúde humana^(52)^. Portanto, faz-se necessário que cada vez mais novos estudos sejam realizados para ampliar o conhecimento sobre a necessidade do cuidado espiritual e religioso em cenários vulnerados, os quais comprometem o bem-estar de crianças, adolescentes e suas famílias. Também são necessários estudos que ampliem as evidências positivas de oferecimento do suporte espiritual pelos profissionais de saúde.

Desse modo, ao compreender as complexidades dos grupos vulneráveis e a espiritualidade e a religiosidade como ferramentas que podem ser utilizadas, é necessário implementar e utilizar tais subsídios por e para essas populações. Por sua vez, através da implementação do cuidado espiritual nos setores da educação e saúde, profissionais poderão planejar e adotar estratégias para assistir tais grupos.

### Limitações do estudo

O quantitativo de bases de dados pesquisadas pode ter contribuído para limitar o acesso a outros dados. Outro fator limitante foi o contexto pesquisado, no qual foi identificado somente um estudo brasileiro. Acredita-se que este artigo contribua para o desenvolvimento de futuras pesquisas, sobretudo na América Latina, tendo em vista que somente dois artigos foram encontrados.

### Contribuições para as áreas da enfermagem, saúde, ou políticas públicas

O estudo desenvolvido permite identificar as potencialidades e fragilidades quanto à espiritualidade e religiosidade de crianças, adolescentes e suas famílias em um contexto vulnerável, o que possibilita a implementação de medidas na formação dos profissionais de saúde para que haja uma abordagem espiritual/religiosa ao paciente, no intuito de garantir uma assistência holística e integral.

## CONSIDERAÇÕES FINAIS

A presente revisão de escopo contribuiu para demonstrar que existe influência da religiosidade e espiritualidade na vida de crianças, adolescentes e familiares, e isso foi retratado como fator de proteção, fonte de conforto e ferramenta de resiliência no enfrentamento das adversidades em um contexto vulnerável.

Portanto, investigar como a presença da espiritualidade e da religiosidade pode influenciar o processo saúde doença torna-se primordial para a execução de uma assistência integral à criança, adolescente, família e coletividade, haja vista a necessidade de considerar o contexto biopsicossocial e espiritual em que o indivíduo está inserido.
